# *Chlamydomonas* IC97, an intermediate chain of the flagellar dynein f/I1, is required for normal flagellar and cellular motility

**DOI:** 10.1128/msphere.00558-24

**Published:** 2024-11-27

**Authors:** Ryosuke Yamamoto, Yui Tanaka, Shunsuke Orii, Kogiku Shiba, Kazuo Inaba, Takahide Kon

**Affiliations:** 1 Department of Biological Sciences, Graduate School of Science, Osaka University, Osaka, Japan; 2 Shimoda Marine Research Center, University of Tsukuba, Shizuoka, Japan; California State University Fullerton, Fullerton, California, USA

**Keywords:** *Chlamydomonas*, eukaryotic flagella (cilia), IDA f/I1, IC97 (DII6/FAP94), CASC1 (LAS1/CFAP94/DNAI7), FAP120

## Abstract

Motile flagella (also called “motile cilia”) play a variety of important roles in lower and higher eukaryotes, including cellular motility and fertility. Flagellar motility is driven by several species of the gigantic motor-protein complexes, flagellar dyneins, that reside within these organelles. Among the flagellar-dynein species, a hetero-dimeric dynein called “IDA f/I1” has been shown to be particularly important in controlling the flagellar waveform, and defects in this dynein species in humans cause ciliopathies such as multiple morphological abnormalities of the flagella and asthenoteratozoospermia. IDA f/I1 is composed of many subunits, including two HCs (HCα and HCβ) and three ICs (IC140, IC138, and IC97), and among the three ICs of IDA f/I1, the exact molecular function(s) of IC97 in flagellar motility is not well understood. In this study, we isolated a *Chlamydomonas* mutant lacking IC97 and analyzed the phenotypes. The *ic97* mutant phenocopied several aspects of the previously isolated IDA-f/I1-related mutants in *Chlamydomonas* and showed slow swimming compared to the wild type but retained the ability to phototaxis. Further analysis revealed that the mutant had low flagellar beat frequency and miscoordination between the two (*cis*- and *trans*-) flagella. In addition, the mutant cells swam in a comparatively straight path compared to the wild-type cells. Taken together, our results highlight the importance of proper assembly of IC97 in the IDA-f/I1 complex for the regulation of flagellar and cellular motility in *Chlamydomonas* and provide valuable insights into both the molecular functions of IC97 orthologs in higher eukaryotes and the pathogenetic mechanisms of human ciliopathies caused by IDA-f/I1 defects.

**IMPORTANCE:**

IDA f/I1 is a hetero-dimeric flagellar dynein that is particularly important for the regulation of flagellar waveform and whose defects are associated with human ciliopathies. IC97 is an evolutionarily conserved intermediate chain of IDA f/I1, but the detailed molecular functions of IC97 in flagellar motility have not been elucidated. In this study, mutational and biochemical analyses of the previously uncharacterized *Chlamydomonas ic97* mutant revealed that IC97 is required for both the normal flagellar and cellular motility. In particular, IC97 appears to play an important role in both the control of flagellar beat frequency and the coordination between the two (*cis*- and *trans*-) flagella in *Chlamydomonas*. Our results provide important insights into the regulation of IDA-f/I1 activity by IC97 and the pathogenetic mechanisms of human ciliopathies caused by IDA-f/I1 defects.

## INTRODUCTION

Motile flagella (also interchangeably referred to as “motile cilia”) are organelles present in many eukaryotes and play important roles in various aspects of these organisms. For example, in unicellular eukaryotes such as *Euglena* and *Trypanosoma*, motile flagella are important for environmental sensing and cellular motility (1). In multicellular higher eukaryotes, including humans, these organelles also function in normal fertility and development, and their defects cause various symptoms (e.g., hydrocephalus, bronchitis, and situs inversus), which are collectively referred to as “ciliopathies” (2, 3). Due to the importance of motile flagella, the mechanism(s) by which these organelles generate movement has been the focus of many researchers for decades.

**Fig 1:**
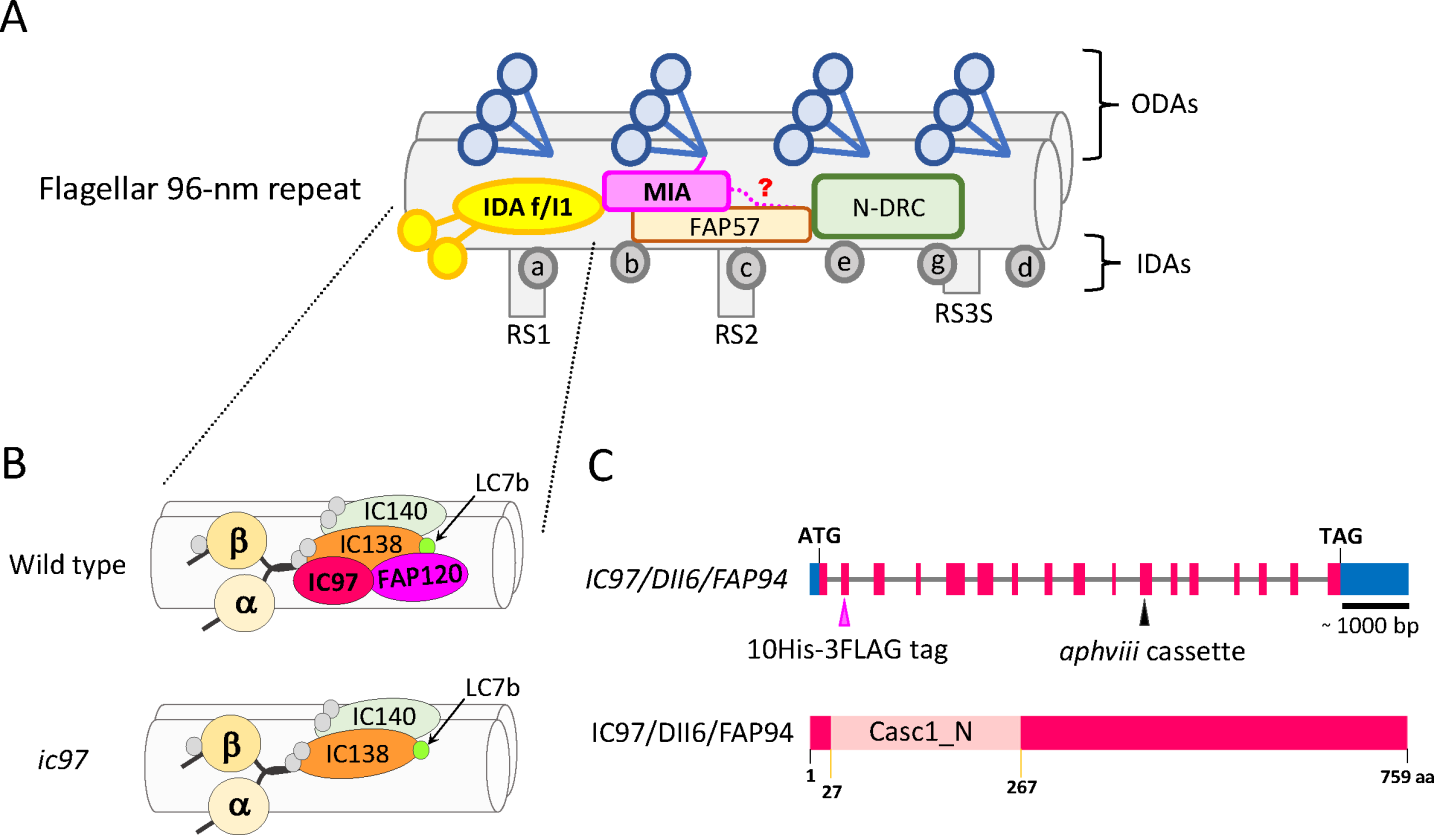
Diagrams of the *Chlamydomonas* flagellar 96-nm repeat, IDA f/I1, and the *IC97* gene/IC97 protein. (**A**) Diagram of the classical *Chlamydomonas* flagellar 96-nm repeat. The focus of this study is the hetero-dimeric IDA species, IDA f/I1, located on the left side of the diagram. The small gray circles in the diagram indicate other monomeric IDA species (IDAs a, b, c, d, e, and g). Through the modifier-of-inner-arms (MIA) complex (mainly composed of the FAP100 and FAP73 heterodimer) (16), IDA f/I1 is linked to the flagellar doublet microtubule, ODA, and further to the N-DRC via the N-terminal half of the FAP57 homodimer (17). The MIA complex is thought to mechanochemically coordinate the activity of these important flagellar structures (18, 19). FAP57 was biochemically predicted as a potential member of the MIA complex in the original description of the complex (16) and was shown to link IDA f/I1 and the MIA complex to the N-DRC (17). The actual N-terminal end of the MIA complex, which was not modeled in the previous report (17) and which is likely to further interact with the FAP57 homodimer/N-DRC, remains to be determined by future studies. RS1/2, radial spoke 1/2; RS3S, radial spoke 3 stump. (**B**) Diagram of the IDA-f/I1 structural model in the wild type and the *ic97* mutant. The model is based on this study and previous reports (12, 20–22). *Chlamydomonas* IDA f/I1 has two HCs (HCα and HCβ), three ICs (IC140, IC138, and IC97), and several LCs including FAP120 and LC7b (Fig. S1A). The *ic97* mutant lacks IC97 and FAP120 (also see the main text and Fig. 2A and D). (**C**) Structural models of the *Chlamydomonas IC97/DII6/FAP94* gene and IC97/DII6/FAP94 protein. The gene structure is based on the Phytozome *Chlamydomonas* v5.6 (https://phytozome-next.jgi.doe.gov/info/Creinhardtii_v5_6) and the previous study [NCBI: ACN22075.1 (23)] (blue, 5*'*/3*'* UTR; red, exon; gray, intron). The hypothetical insertion site of the *aphviii* cassette in the original CLiP *ic97* strain (LMJ.RY0402.067788) (24) and the insertion site of the10His-3FLAG tag in the rescued strain (*ic97; IC97:10His-3FLAG-TG*) are also shown. The 10His-3FLAG tag was inserted into the second exon of the *IC97* gene due to the availability of a suitable restriction site (NheI) for protein tagging. The position of the Casc1_N domain in the IC97 protein (NCBI: ACN22075.1 [23]) was predicted by the SMART analysis (http://smart.embl-heidelberg.de/ [25]).

Through extensive biochemical and biophysical studies, researchers eventually showed that flagellar motility is driven by gigantic motor-protein complexes called “flagellar dyneins” that are anchored to the doublet microtubules inside the flagella (4, 5). Flagellar dyneins can be divided into two major groups: outer-arm dyneins (ODAs) and inner-arm dyneins (IDAs) (6, 7) (Fig. 1A). While ODAs are necessary for flagella to generate strong propulsive force and high beat frequency, IDAs are important for controlling the flagellar waveform (8). Furthermore, IDAs can be subdivided into seven major species, designated “IDA a” to “IDA g.” Among these seven IDA species, only IDA f (or I1, hereafter referred to as “f/I1,” Fig. 1A and B; Fig. S1A and B) is a hetero-dimeric dynein, while the other six species are monomeric dyneins (7, 9–11). Previous studies have shown that IDA f/I1 is particularly important for the regulation of flagellar waveform (12, 13), and its defects are associated with ciliopathies such as multiple morphological abnormalities of the flagella and asthenoteratozoospermia in humans (14, 15). However, despite its critical role in flagellar motility, many questions remain about how IDA-f/I1 activity is regulated.

The regulatory mechanism(s) of IDA-f/I1 activity, as well as the subunit composition of IDA f/I1, have been mainly studied using a green flagellated alga, *Chlamydomonas reinhardtii* as a model organism (1, 12). *Chlamydomonas* has two (*cis*- and *trans*-) motile flagella, and studies of the purified IDA f/I1 from these flagella revealed that IDA f/I1 is composed of two heavy chains [HCs: HCα (DHC1) and HCβ (DHC10)], three intermediate chains (ICs: IC140, IC138, and IC97), and many light chains including FAP120 and LC7b (12, 26, 27) (Fig. 1B; Fig. S1A). Among these various subunits, HCα and HCβ, as well as IC140 have been shown to be indispensable for the assembly and attachment of IDA f/I1 to the flagellar microtubules (28–31), and IC138 has been hypothesized to function as a “hub” for the control of IDA-f/I1 activity through phosphorylation (20, 32, 33). In contrast to these subunits, the exact molecular function of IC97 is not well understood, in part due to the unavailability of an *ic97* mutant, although it has been proposed that IC97 functions in concert with IC138 and forms a regulatory sub-complex with IC138, FAP120, and LC7b to control IDA-f/I1 activity (Fig. 1B; Fig. S1A) (23, 33).

In this study, we isolated and analyzed a novel *Chlamydomonas* mutant with a defect in the *IC97* gene. The mutant completely lacked IC97 in the flagella and showed slow swimming compared to the wild type. Interestingly, an incomplete IDA-f/I1 complex containing two other ICs (IC140 and IC138) could be assembled in the flagella of this mutant, indicating that IC97 is dispensable for flagellar assembly and attachment of IDA f/I1. In contrast, FAP120, an LC of IDA f/I1, was absent or greatly reduced in the mutant flagella, suggesting that IC97 is important for flagellar localization of this LC. We also found that the *ic97* mutant retained the ability to phototaxis but had both low flagellar beat frequency and miscoordination between the two flagella. Furthermore, the mutant cells swam in a comparatively straight path compared to the wild-type cells, reminiscent of the “*ida*” mutants of *Chlamydomonas*. These results strongly suggest that proper regulation of flagellar motility and coordination by IDA f/I1 requires the assembly and function of IC97 and FAP120 in this dynein species and that loss of these two subunits is sufficient to cause aberrant flagellar and cellular motility.

## RESULTS AND DISCUSSION

### IC97 is dispensable for flagellar IDA-f/I1 assembly but required for flagellar localization of FAP120

*Chlamydomonas* IC97 (also known as DII6 or FAP94) is a conserved 759-amino acid protein (23) and has the “Casc1_N domain” in the N-terminal half of the molecule (Fig. 1C; Fig. S1C and S2). The higher-eukaryotic ortholog [cancer susceptibility candidate 1 (CASC1); Fig. S1B, D, and Fig. S2] of *Chlamydomonas* IC97 has been associated with lung tumors (34, 35), but its exact molecular function is not fully understood.

**Fig 2:**
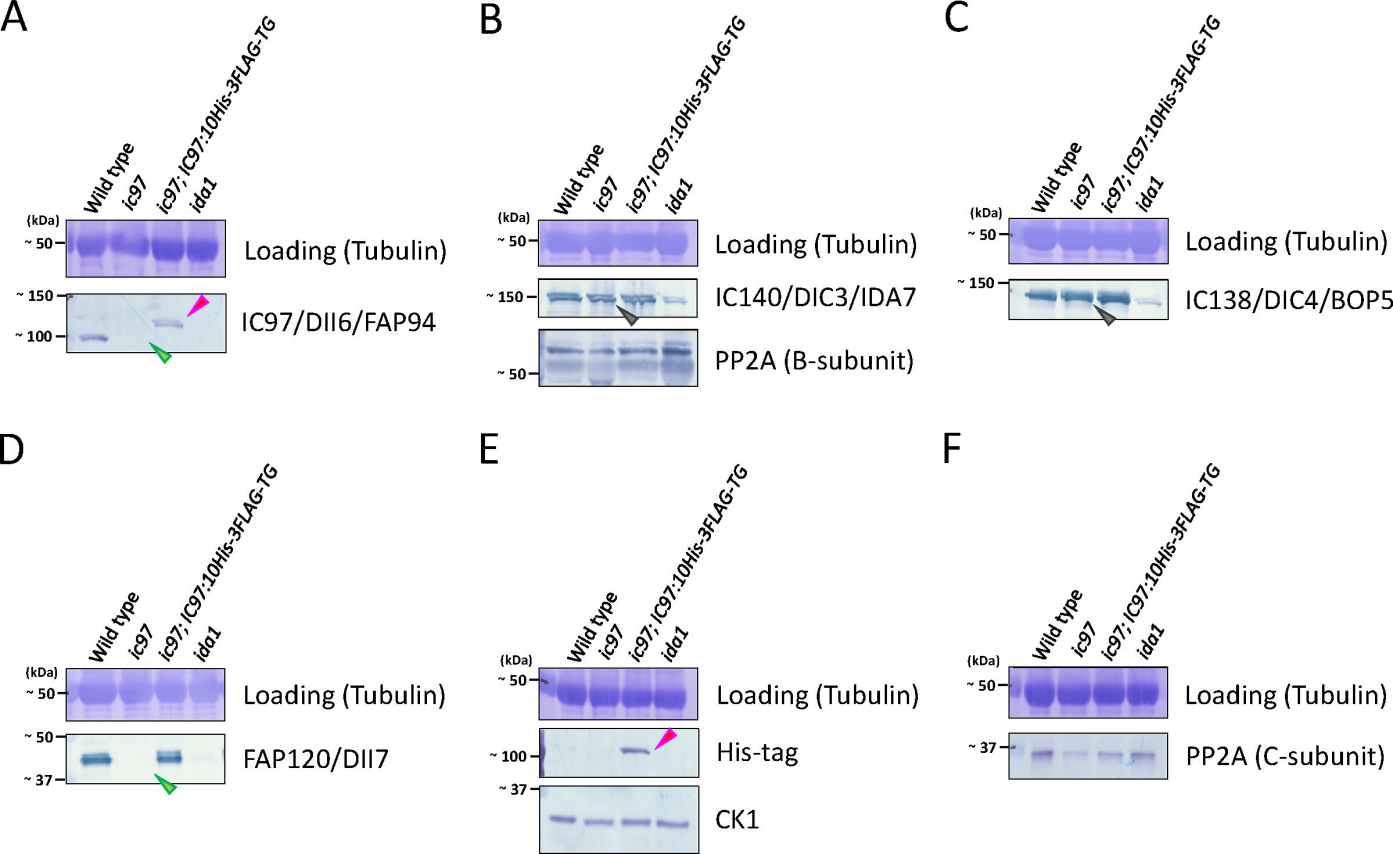
The *ic97* mutant lacks IC97 and FAP120 proteins in the flagellar axoneme. (A–F) Western blottings of axonemes from the wild type, the *ic97* mutant, the rescued strain, and *ida1* using antibodies against IDA-f/I1-related proteins. IC138 and IC140 are present in the *ic97* axoneme [gray arrowheads in (**B**) and (**C**)], whereas IC97 and FAP120 are absent/greatly reduced [green arrowheads in (**A**) and (**D**)]. These losses are restored in the rescued strain [(**A**) and (**D**)], and the rescued strain also has a slightly larger IC97 with a 10His-3FLAG tag [IC97-10His-3FLAG; red arrowheads in (**A**) and (**E**)]. Sometimes, for unknown reasons, we observed a few bands in the IC140/IC138/IC97 blots, but these bands may represent protein degradation and/or some modification(s). The amount of the C- (catalytic-) subunit of PP2A in the *ic97* flagellar axoneme varied with sample preparation, ranging from reduced [(**F**) in this figure] to apparently normal. The reason for this variability is unclear, but it is possible that the C-subunit of PP2A in *ic97* flagella is unstable and easily degraded during sample preparation and/or that the amount of this subunit in the flagella actually varies during the life cycle of the *ic97* mutant.

**Fig 3:**
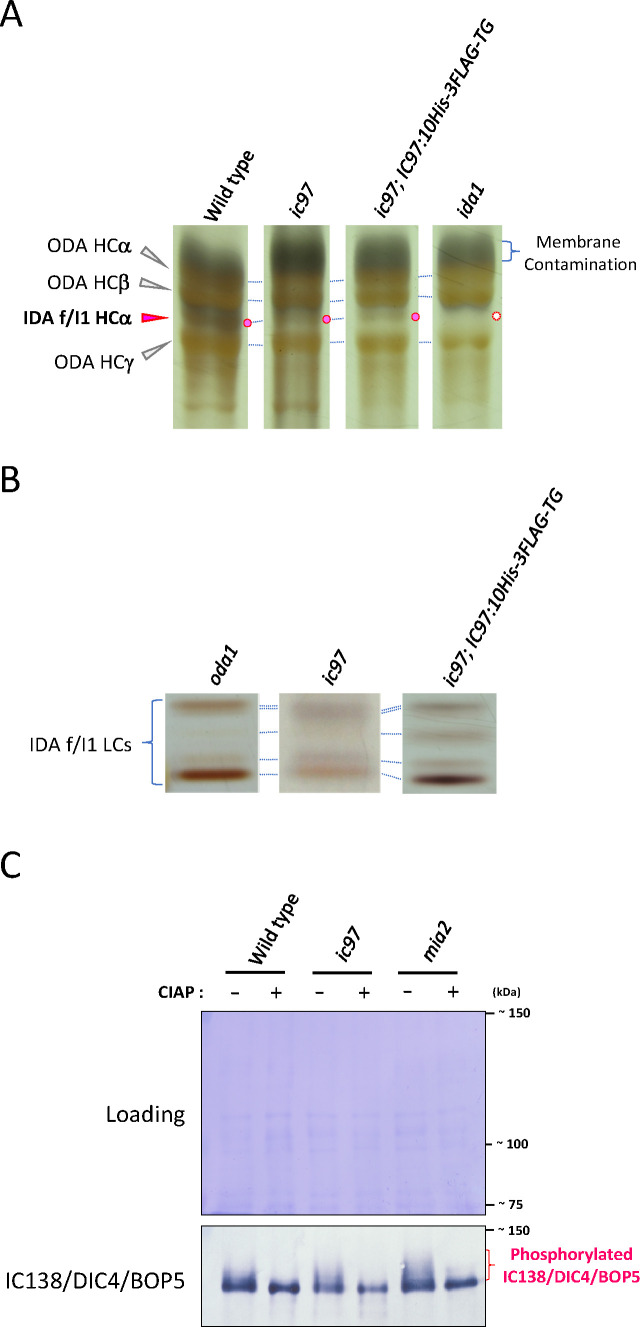
The abundance of IDA-f/I1-HCs/LCs is relatively normal, but the phosphorylation of IC138 is apparently increased in the *ic97* mutant. (**A**) Urea-gel PAGE of dynein HCs from axonemes of the wild type, the *ic97* mutant, the rescued strain, and *ida1*. The *ic97* mutant has the HCα of IDA f/I1 (red circle), which is missing in *ida1* (white circle). The other HC (HCβ) of IDA f/I1 overlaps with the HCγ of ODA and cannot be observed in this gel. The relative position of the lanes has been adjusted for presentation, and all lanes are from the same gel. (**B**) SDS-PAGE patterns of LCs (~10 kDa) of purified IDA f/I1 from axonemes of *oda1*, the *ic97* mutant, and the rescued strain using Uno-Q ion-exchange chromatography. For this analysis, we used *oda1* instead of the wild type to facilitate the separation of IDA f/I1. There is no major difference in LC (~10 kDa) composition between these strains. All lanes are from different gels, and the development time of silver staining, and the contrast and/or brightness of the images were adjusted to clearly observe the LCs on each gel. (**C**) Western blotting of the wild type, the *ic97* mutant, and *mia2* axonemes using the IC138 antibody. Axonemes were treated with or without calf intestinal alkaline phosphatase (CIAP) for ~1 hour prior to analysis. The *mia2* mutant has been shown to have hyperphosphorylated IC138 in flagella (16, 36). The phosphorylation state of IC138 in the *ic97* mutant appears to be intermediate between the wild type and *mia2*.

To understand the molecular function of IC97, we first isolated a novel *ic97* mutant of *Chlamydomonas* (the original strain [LMJ.RY0402.067788] was obtained from the CLiP library [24]; Fig. 1C; Table S1) and biochemically analyzed the composition of IDA f/I1 in the mutant flagella. Based on the western-blotting analysis, we did not detect any IC97 signal in the flagellar sample of the *ic97* mutant (Fig. 2A), strongly suggesting that this is a null mutant. Interestingly, other major components of IDA f/I1, including the two HCs (HCα and HCβ) and other ICs (IC140 and IC138), are present in the mutant flagella (Fig. 2B and C, 3A). This indicates that IC97 is dispensable for flagellar assembly of these IDA-f/I1 subunits, in agreement with the previous report (21). We also did not observe any major defects in the LC composition of the mutant flagella (data not shown), except that the amount of one LC, FAP120, was greatly reduced or missing (Fig. 2D and 3B). The amount of flagellar FAP120 was restored in the rescued strain (*ic97; IC97:10His-3FLAG-TG*) expressing the epitope-tagged IC97 protein (IC97-10His-3FLAG) (Fig. 1C, Fig. 2A and E; Fig. S1C and S2; Table S1). These results are consistent with the recent structural analysis, showing that IC97 and FAP120 are localized in close proximity to each other in the IDA-f/I1 complex (Fig. S1A) (17).

In addition, the phosphorylation of IC138 (32, 37) appeared to be increased in *ic97* flagella compared to the wild type but not as hyperphosphorylated as in the previously characterized *mia2* mutant (Fig. 3C), in which IC138 is reported to be highly phosphorylated, and IDA f/I1 is inactivated (16, 36). As in *mia2* flagella (16), CK1 and PP2A, the kinase and phosphatase hypothesized to regulate IC138, were present in *ic97* flagella, although the amounts appeared to vary slightly between cultures (Fig. 2B, E, and F) (38, 39). Based on our results, the loss of IC97 in flagella seems to have only a limited effect on the flagellar localization of CK1 and PP2A but may have a large effect on the relative position(s) between these enzymes and IC138 (substrate) in the axonemes. Our results also suggest that the defects in flagellar and cellular motility observed in the *ic97* mutant, which we will describe in this report, may be caused, at least in part, by the increased IC138 phosphorylation and inactivated IDA f/I1 in *ic97* flagella.

### The *ic97* mutant retains the ability to respond to phototactic stimuli

**Fig 4:**
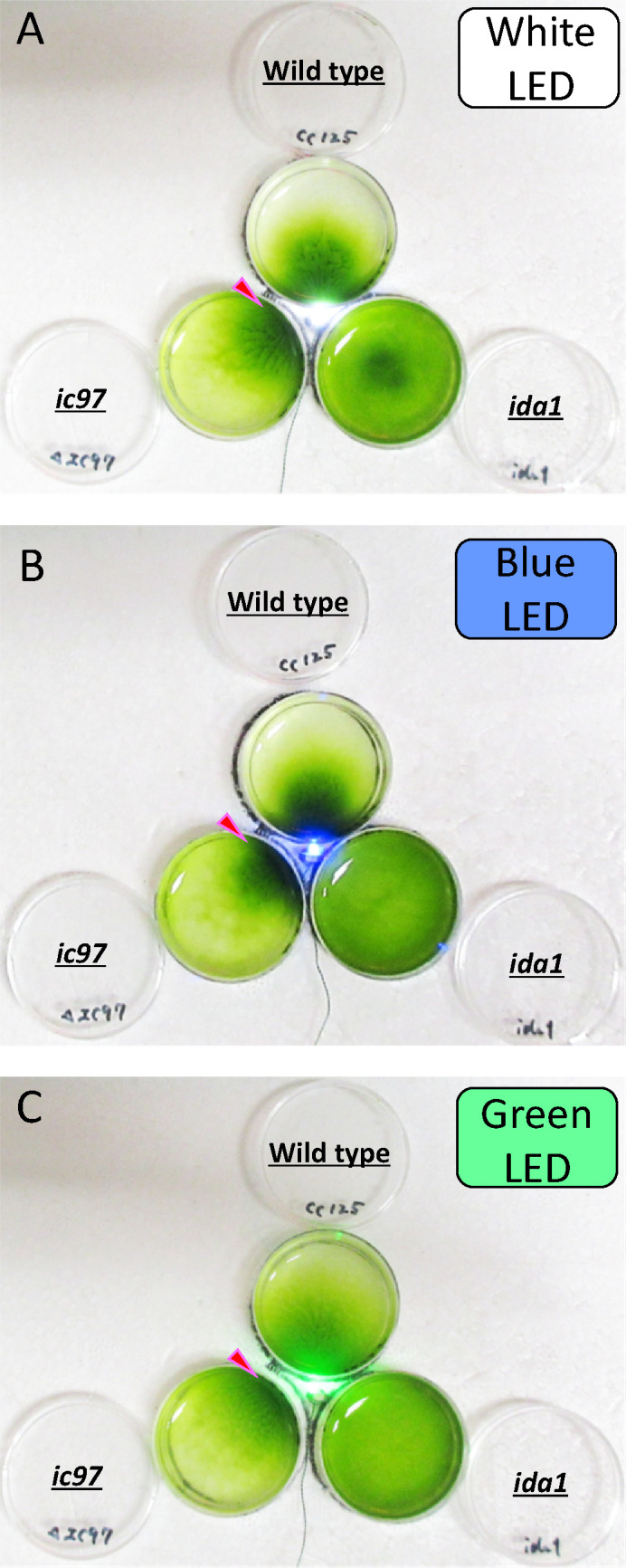
The *ic97* mutant retains the ability to show phototaxis. (A–C) Phototaxis dish assay of the wild type, the *ic97* mutant, and *ida1*. Prior to the assay, the strains were exposed to red light for at least 15 minutes and then to white (**A**), blue (**B**), or green (**C**) LED for 30 minutes. The *ic97* mutant showed the ability to phototaxis to all light colors tested (red arrowheads). The *ida1* cells appeared to accumulate at the center of the dish under the presence of white light for unknown reason(s) (**A**), but the cells may have condensed in the slightly sunken part of the dish, and/or many cells may have accidentally floated away from the rim of the dish at the time the photograph was taken. Brightness and contrast of photographs were adjusted for presentation.

IDA-f/I1 defects in *Chlamydomonas* have often been associated with an inability to display phototaxis (16, 20, 40). Therefore, we next examined the phototactic response of the *ic97* mutant using the dish assay (Fig. 4A through C). Unlike *mia2* (Fig. 1A; Fig. S1A; Table S1) (16, 20, 36) or *ida1* (28, 41), which has a mutation in one (HCα) of the two IDA-f/I1 HCs and completely lacks flagellar IDA f/I1 (Table S1), the *ic97* mutant showed normal phototaxis to all light colors tested (white, green, and blue) (Fig. 4A through C). This phenotype is similar to that of the *bop5* mutant (Table S1), which carries a mutation in the *IC138* gene and has greatly reduced amounts of IC97, FAP120, and LC7b in the flagella (Table S1) (20, 32, 33). Our result suggests that IC97 and FAP120, which are both deficient in *ic97* flagella, are not involved in the signaling pathway of *Chlamydomonas* phototaxis.

### The *ic97* mutant exhibits slow and aberrant swimming

**Fig 5:**
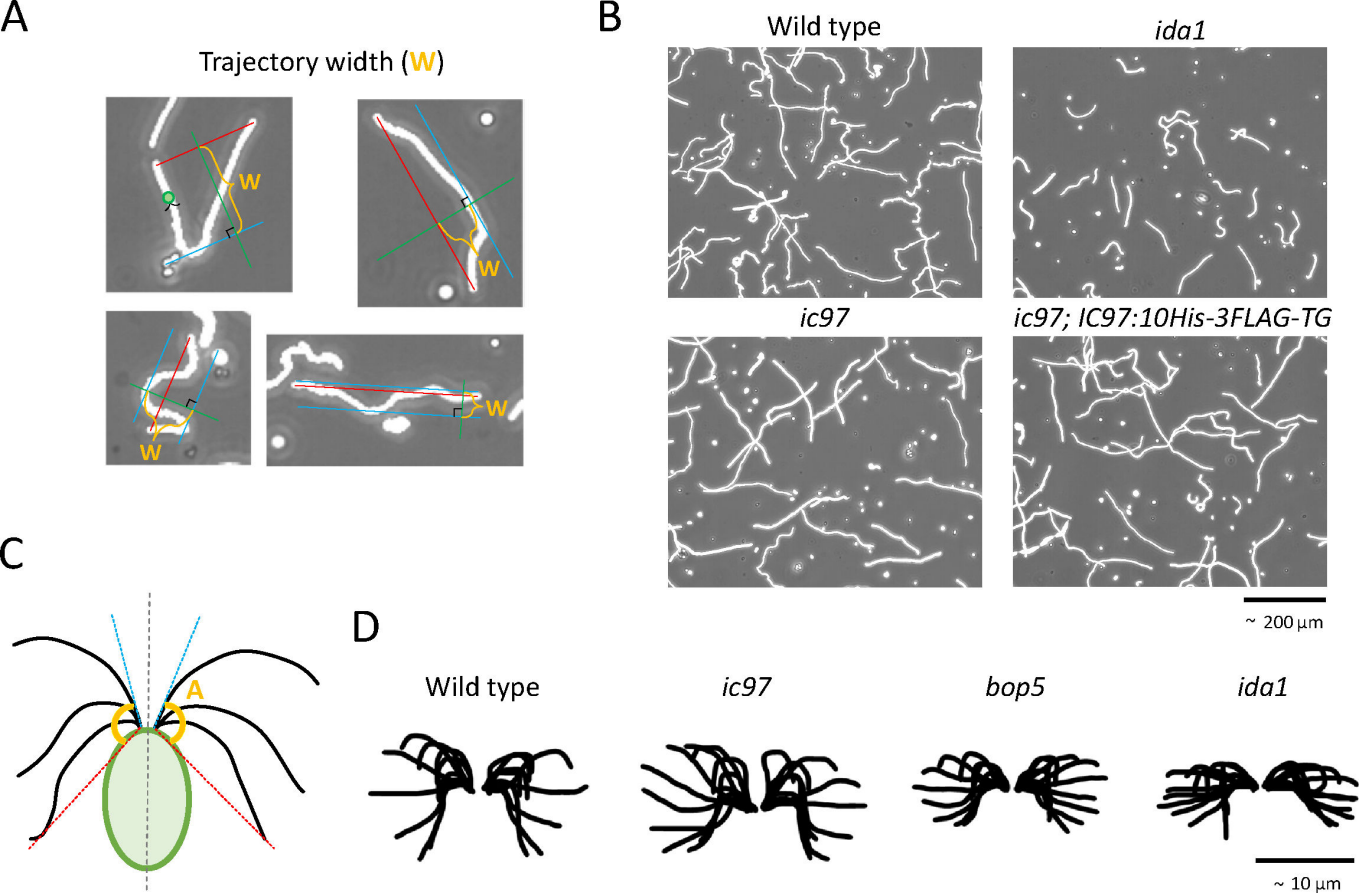
The *ic97* mutant has altered cellular motility, but the flagellar waveform is relatively similar to the wild type. (**A**) The approximate maximum width of the *Chlamydomonas* swimming path per 1 second (trajectory width) was measured in this study and is summarized in Table 1. This value becomes smaller when cells swim in a relatively straight path, while it becomes larger when cells swim in paths with turns and changes of direction. (**B**) The stacked images of the swimming path of *Chlamydomonas* cells for 1.5 seconds. Like the *ida1* cells, the *ic97* mutant cells swim comparatively straighter paths than the wild-type and rescued-strain cells. (**C**) Schematic of the flagellar-amplitude-calculation method. The approximate angle from the tangent line (blue) at the flagellar upstroke to the action line (red) at the flagellar downstroke relative to the long axis of the cell was measured to compare the amplitude of the flagellar waveform during swimming and is summarized in Table 1. (**D**) The traced patterns of the flagellar waveform of the wild type, the *ic97* mutant, *bop5,* and *ida1*. While *bop5* and *ida1* have smaller flagellar amplitudes like other typical *ida* mutants (Table 1) (8, 20), the flagellar waveform of the *ic97* mutant was relatively similar to that of the wild type. The approximate flagellar waveform was traced with PowerPoint (Microsoft) using the images from the 500-fps movies.

Next, to gain insight into the molecular function(s) of IC97 in *Chlamydomonas* swimming, we carefully observed the cellular swimming phenotypes of the *ic97* mutant. Unlike the wild-type cells, the *ic97* mutant swam relatively slowly and smoothly without many turns or changes in direction (Fig. 5A and B; Table 1), reminiscent of many “*ida*” mutants of *Chlamydomonas* that have defects in IDA-related proteins (41, 42). The swimming velocity of the *ic97* mutant (~121.4 µm/s) was slower compared to the wild type (~161.2 µm/s), and this slow swimming phenotype was recovered in the rescued strain expressing the IC97-10His-3FLAG protein (Fig. 2A and E; Fig. S1C and S2; Table 1).

**TABLE 1:** Swimming parameters calculated/estimated in this study^*i*^

	Wild type	*ic97*	*ic97; IC97:10His-3FLAG-TG*	*bop5*	*ida1*
Swimming velocity (μm/s)^*a*^	161.2 ± 18.0	121.4 ± 15.0^*b*^	159.3 ± 16.1	120.5 ± 9.6	79.4 ± 8.9
Flagellar beat frequency (Hz)^*c*^	71.3 ± 4.7^*d*^	44.9 ± 5.4^*b*^	61.0 ± 8.8	49.5 ± 12.5	54.5 ± 10.5
Flagellar amplitude (°)^*e*^	137.7 ± 6.1	132.1 ± 5.9	132.4 ± 7.8	119.4 ± 7.1	101.5 ± 5.0
Trajectory width (μm)^*f*^	19.4 ± 10.9	14.0 ± 8.6^*b*^	17.8 ± 9.4	9.0 ± 5.3	9.0 ± 4.7
Flagellar coordination ratio (%)^*g*^ (live cell)	93.6	65.3^*b*^	89.6	67.3	72.0
Flagellar coordination ratio (%)^*g*^^,^^*h*^ (cell model)	62.3	61.2	(Not measured)	53.7	59.8

^*a*^ For each strain, data from two independent experiments (*n* = 100) were combined to calculate the mean swimming velocity (total *n* = 200). Swimming velocity was calculated from the swimming length of cells per second, and the length was manually tracked using Image J.

^*b*^ *P* < 0.05 vs the wild type (Tukey’s test or chi-squared test was used to compare swimming velocity, trajectory width, flagellar beat frequency and amplitude, or flagellar coordination ratio).

^*c*^ To estimate the flagellar beat frequency of a specific strain, we first counted the frame numbers for a beat cycle using the 500-fps movies, manually calculated the average beat frequency of a given flagellum, and then averaged the beat frequency of multiple flagella to estimate the beat frequency of the specific strain as a whole. The beat frequency of ~20–40 flagella was averaged to estimate the beat frequency of the specific strain. We could not distinguish between *cis*- and *trans*-flagella in this analysis.

^*d*^ In our analysis, the beat frequency of the wild-type strains varied slightly depending on the strain and culture (CC124: ~71 Hz [in this table], CC1245: ~61 Hz). Such variations in the beat frequency of flagella have been also reported previously and likely represent a slight variability in the flagellar length of the cells analyzed (43) (see also the Materials and Methods).

^*e*^ To measure the flagellar amplitude, we measured the approximate angle of the flagellar trajectory (see Fig. 5C) using the 500-fps movies. For this analysis, we selected the synchronized flagella as much as possible because the flagellar amplitude was often different in the desynchronized flagella. First, we calculated the average flagellar angle (Fig. 5C) for several beat cycles of a given cell and then averaged the angles of multiple cells (9–20 cells) to estimate the flagellar amplitude of the specific strain. We could not distinguish between *cis*- and *trans*-flagella in this analysis.

^*f*^ The width of the trajectory advanced by cells (*n* = ~100, see Fig. 5A) per second was measured.

^*g*^ To calculate the flagellar coordination ratio, the number of times the two flagella were in phase (aligned) or out of phase (not aligned) was manually counted from the 500-fps movies when one flagellum was approximately perpendicular to the long axis of the cell (see Fig. 6B). The percentage of coordination was calculated from the several hundred beat counts. We could not distinguish between *cis*- and *trans*-flagella in this analysis.

^*h*^ For the cell-model analysis, we used independent cultures from the live-cell analysis (upper column).

^*i*^ Values shown are mean ± SD.

### IC97 is required for the generation of the normal flagellar beat frequency

Intrigued by the slow and aberrant swimming phenotype of the *ic97* mutant, we decided to carefully observe the flagellar movement of the *ic97* mutant in order to determine the factor(s) causing this phenotype. First, we found that *ic97* mutants had apparently normal flagellar amplitudes (similar to that of the wild type and significantly wider than that of *ida1*) (Fig. 5C and D; Table 1). Second, we approximately estimated the flagellar beat frequency of the *ic97* mutant from the swimming movie captured by the 500-fps high-speed camera. Interestingly, the *ic97* cells swam with a beat frequency of ~45 Hz, which is lower than that of the wild type (~71 Hz) and the rescued strain (~61 Hz) but more similar to that of the *bop5* (~50 Hz) (Table 1). Given that ODAs have been shown to be particularly important for generating the high beat frequency of flagella (8), the loss of IC97 and FAP120 in both the *ic97* and *bop5* mutants may disrupt or attenuate the mechano-regulatory link between IDA f/I1 and ODAs (16, 44), most likely mediated by the outer-inner dynein linkers between IDA f/I1 and ODA (9, 45), and ultimately cause this low flagellar beat frequency.

### IC97 is important for the proper coordination of two flagella in *Chlamydomonas*

**Fig 6:**
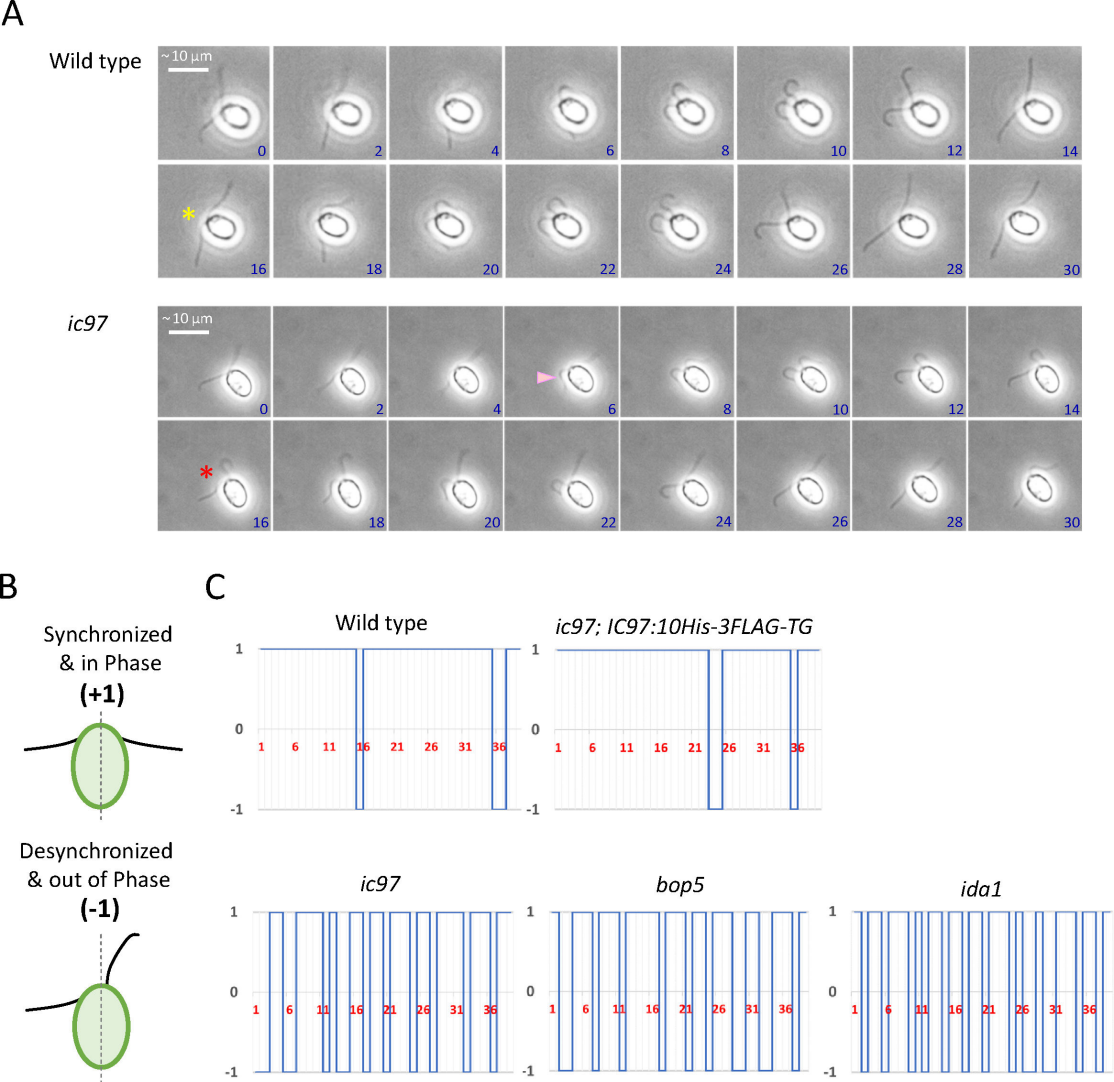
The *ic97* mutant, like the other IDA-f/I1-related mutants, has aberrant flagellar coordination. (**A**) Sequential images of synchronized wild-type flagella (top) and desynchronized *ic97* flagella (bottom). The numbers in blue in the images indicate the time stamp in milli-seconds. While the two wild-type flagella are in phase most of the time, the mutant flagella are often out of phase and return to phase after one or two cycles of desynchronization [compare the beat phase of wild-type and *ic97* flagella (yellow and red asterisks, respectively)]. In the *ic97* mutant, we observed that the two flagella often begin to desynchronize at the beginning of the effective or recovery stroke, and in this figure, the desynchronization occurs at the beginning of the recovery stroke of the faster-beating flagellum (pink arrowhead). The brightness and contrast of the images have been adjusted to make the beating flagella clearly visible. (**B**) To calculate the coordination ratio of two flagella, flagella were defined as “in phase” if both were approximately perpendicular to the long axis of the cell (top) and “out of phase” if only one flagellum was approximately perpendicular to the long axis (bottom) during the beat cycle. The calculated coordination ratio is summarized in Table 1. To binarize the synchronization/desynchronization of flagella, (+1) was defined as flagella in phase (top), and (−1) was defined as flagella out of phase (bottom), and examples of binarized flagellar coordination are shown in panel C. (**C**) The binarized coordination/miscoordination of two flagella analyzed in the wild type, the *ic97* mutant, the rescued strain, *bop5,* and *ida1*. For each strain, flagella were selected from a cell with a typical phenotype and plotted in the step charts as [(in phase = 1)/(out of phase = −1)] during beat cycles. Flagella of the IDA-f/I1-related mutants often showed flagellar miscoordination. The numbers in red represent the number of flagellar beat cycles. Several beat cycles, where we could not observe the flagella/distinguish coordination or the flagella beat in particularly abnormal shapes, were omitted and other observable beat counts were continuously combined.

In addition to the low beat frequency, we found that the coordination between *cis*- and *trans*-flagella is abnormal in the *ic97* mutant. In the wild-type cell, the two flagella are synchronized and beat in the same phase most of the time (Fig. 6A). Because the *trans*-flagellum beats at a slightly higher frequency than the *cis*-flagellum (46), the two flagella sometimes go out of phase even in the wild type, but in this case, the two flagella quickly return to the same phase. In the *ic97* mutant, however, the frequency with which the two flagella go out of phase and desynchronize was much higher than in the wild type (Fig. 6B and C; Table 1). The switch from synchronization to desynchronization occurred abruptly in *ic97* flagella, with one or two cycles of desynchronization followed by a return to synchronization (Fig. 6A through C). Although, in these analyses, we could not definitively distinguish between *cis*- and *trans*-flagella because of the difficulty in identifying eyespots in the recorded movies, we also noticed that the switch from synchronization to desynchronization often occurred at the beginning/early phase of both the effective and recovery strokes of the *ic97* flagella (Fig. 6A). The miscoordination between the two flagella was also frequently observed in both live cells and cell models of the IDA-f/I1-related mutants (*ic97*, *bop5,* and *ida1*) (Fig. 6C; Table 1) and may be one reason why the IDA-f/I1-related mutants show reduced flagellar beat frequency compared to the wild type (Table 1; [16, 41]). Since IDA f/I1 is associated with many important flagellar structures, including the ODAs, the N-DRC, and the doublet microtubule (Fig. 1A) (12, 16, 17, 47), the physical interaction(s) between IDA f/I1 and these structures are likely to be important for flagellar coordination both *in vivo* (in live cells) and *in vitro* (in cell models), and the loss of IC97 and FAP120, in combination with the increased phosphorylation of IC138 (Fig. 3C), may disrupt or alter the interaction(s) between IDA f/I1 and these flagellar structures, ultimately causing the defects in flagellar and cellular motility observed in the *ic97* mutant.

### Conclusions

In this short report, we have analyzed the previously uncharacterized *Chlamydomonas* mutant lacking IC97, an intermediate chain of the flagellar dynein IDA f/I1. IC97 was found to be essential for the flagellar localization of the IDA-f/I1 LC, FAP120. The *ic97* mutant showed some characteristic features, including the aberrant swimming trajectory, altered flagellar beat frequency and coordination while retaining the ability to phototaxis. The results concisely presented in this paper demonstrate that *Chlamydomonas* IC97 is required for both normal flagellar and cellular motility, and should provide important insights into the molecular function of CASC1, the human ortholog of IC97 and a candidate protein for ciliopathies.

## MATERIALS AND METHODS

### *Chlamydomonas* strains, cell culture, and isolation of flagella/axonemes

The 137c-derived strains, including CC124, CC125, and CC1245 (progeny from crosses of CC124 and CC125 [22]), were used as *Chlamydomonas* wild-type strains. These strains were considered wild type as long as they exhibited wild-type motility/phenotypes despite the unexpectedly large genetic variance recently reported in these strains (48–50). The mutant strains used in this study are summarized in Table S1. Cells were harvested in the solid or liquid TAP medium (51) under constant light or light/dark cycles (12/12 or 16/8 hours). Isolation of flagella from cells was performed by the dibucaine method, followed by detergent treatment to obtain axonemes (52). Calf-intestinal alkaline phosphatase (CIAP) treatment of axonemes (32, 36) was performed by adding excess amounts (>1 unit per 1 µg protein) of CIAP (Promega) to axonemes in HMDENa buffer (30 mM HEPES, 5 mM MgSO_4_, 1 mM DTT, 1 mM EGTA, 50 mM NaCl [pH7.4]) and incubating the axonemes with CIAP for ~1 hour at room temperature.

### Phenotypic rescue of the *ic97* mutant

To rescue the slow swimming phenotype of the *ic97* mutant, we designed a construct (pIC97-10His-3FLAG-Hyg) expressing the epitope-tagged IC97 protein (IC97-10His-3FLAG). The 10His-3FLAG tag was inserted into the second exon at the NheI site of the wild-type *IC97* gene cloned in plasmid “IC97-15” [a generous gift from Drs. Winfield S. Sale (Emory University) and Maureen Wirschell (University of Mississippi)] (23) (Fig. 1C; Fig. S1C and S2). We also incorporated the hygromycin resistance cassette (*aphvii*) (53) into the plasmid at the EcoRI site for screening. Transformation of the *ic97* mutant was performed by electroporation (54), and the hygromycin-resistant colonies were further screened by motility. Finally, the expression of tagged IC97 in the rescued strain (*ic97; IC97:10His-3FLAG-TG*) (Table S1) was confirmed by western-blotting analysis.

### Swimming-velocity measurement

Free swimming of *Chlamydomonas* cells was recorded at 30 fps by the EXILIM EX-100 camera (CASIO) mounted on the Olympus BX50 microscope. The swimming length per second was manually traced by the Image J software (NIH, https://imagej.net/) to calculate the swimming velocity.

### Trajectory-width and flagellar-amplitude measurement

Free swimming of vegetative *Chlamydomonas* cells was recorded at 100–500 fps using the HAS-U2M camera (DITECT) mounted on the Olympus IX71 microscope with a red strobe light. For trajectory-width measurements, the approximate maximum width of the swim path traveled by *Chlamydomonas* cells per second was manually measured with Image J using the 100-fps movies (Fig. 5A). For flagellar-amplitude measurements, the approximate angles of the flagellar trajectory against the flagellar base (Fig. 5C) were manually analyzed with Image J using the 500-fps movies, and flagella in synchronization/phase were primarily selected for measurements.

### Beat-frequency estimation

For beat-frequency estimation, flagella were carefully observed to determine how many frames correspond to one beat cycle using the 500-fps movies, and the number of frames per flagellar beat was manually counted for each flagellum. We calculated the average beat frequency of a given flagellum and further averaged the beat frequency of multiple flagella to estimate the beat frequency for the specific strain as a whole. Our estimated flagellar beat frequency of the *bop5* mutant (Table 1) was lower than the previous report (20), most likely because we primarily estimated the beat frequency of vegetative cells, whereas the previous report calculated the beat frequency of motility-maximized gametic cells, which have better motility than vegetative cells. Furthermore, we observed a variance in the flagellar beat frequency among vegetative cells depending on the culture conditions, and in another independent analysis of the flagellar beat frequency from Table 1, the wild-type beat frequency was ~76 Hz, whereas the *ic97-*mutant beat frequency was ~57 Hz, also significantly lower than the wild type but higher than *oda1* (~30 Hz).

### Measurement of flagellar coordination

To measure the flagellar coordination in *Chlamydomonas* cells, free swimming of the cells was recorded at 500 fps as described in “Trajectory-width and flagellar-amplitude measurement,” above. We manually counted the number of times that the two flagella were in phase (aligned) or out of phase (not aligned) when one flagellum was approximately perpendicular to the long axis of the cell (Fig. 6B) during the beat cycles. Results of typical cells were also binarized (1 = synchronized/−1 = desynchronized; Fig. 6C).

### *Chlamydomonas* cell model

*Chlamydomonas* cell-model experiments were performed as previously described (55, 56). Cells were demembranated with 0.2% IGEPAL CA-630 (Sigma) and reactivated in the reactivation buffer (55, 56) containing 1 mM EGTA (the Ca^2+^ chelator) and 1 mM ATP. Reactivated cells were observed with the Olympus IX71 microscope as described in “Trajectory-width and flagellar-amplitude measurement,” above.

### Phototaxis assay

The dish assay for *Chlamydomonas* phototaxis was performed in phototaxis buffer as previously described (57). Cells were exposed to red light for at least 15 minutes prior to the assay and then exposed to white, green, or blue light-emitting diode (MY-CRAFT) for 30 minutes in a dark room.

### Other methods

Western blotting and SDS-PAGE were performed according to standard methods (58, 59). For western blottings, the following antibodies were used: first antibody (anti-IC97 [23], anti-IC138 [GRI-4T or GRI-5T] [32], anti-IC140 [31], anti-FAP120 [60], anti-CK1 [38], anti-PP2A B-subunit [39], anti-PP2A C-subunit [61], and anti-His [Qiagen]) and second antibody (Goat-anti-Rabbit-HRP and Goat-anti-Mouse-HRP [Invitrogen]). Dynein separation by urea-gel was performed as previously described (55, 62). To purify IDA f/I1 from flagella, a crude high-salt extract containing all flagellar dyneins was separated on an Uno-Q ion-exchange column as described previously (63). Statistical analyses were performed using Tukey’s test (for comparison of swimming velocity, trajectory width, flagellar beat frequency, and amplitude) and chi-squared test (for comparison of flagellar coordination ratio; Table 1).

## AFTER EPUB

*[This article was published on 27 November 2024 with errors in Table 1 and the Fig. 6 legend. The table and legend were corrected in the current version, posted on 11 December 2024.]*

## SUPPLEMENTAL MATERIAL

Supplemental material msphere.00558-24-s0001.pdf Fig. S1 and S2; Table S1.

ASM does not own the copyrights to Supplemental Material that may be linked to, or accessed through, an article. The authors have granted ASM a non-exclusive, world-wide license to publish the Supplemental Material files. Please contact the corresponding author directly for reuse.
