# Supplementary material for: Chlamydomonas IC97, an intermediate chain of the flagellar dynein f/I1, is required for normal flagellar and cellular motility
Source: mSphere. 2024 Nov 27;9(12):e00558-24. doi: 10.1128/msphere.00558-24 (PMC11656769; doi:10.1128/msphere.00558-24)
Supplement: Supplemental material — Fig. S1 and S2; Table S1. [file msphere.00558-24-s0001.pdf]

Supplemental Materials

Supplemental Figure 1. Molecular structures of the IDA-f/I1 complex and IC97 from *Chlamydomonas* and human.

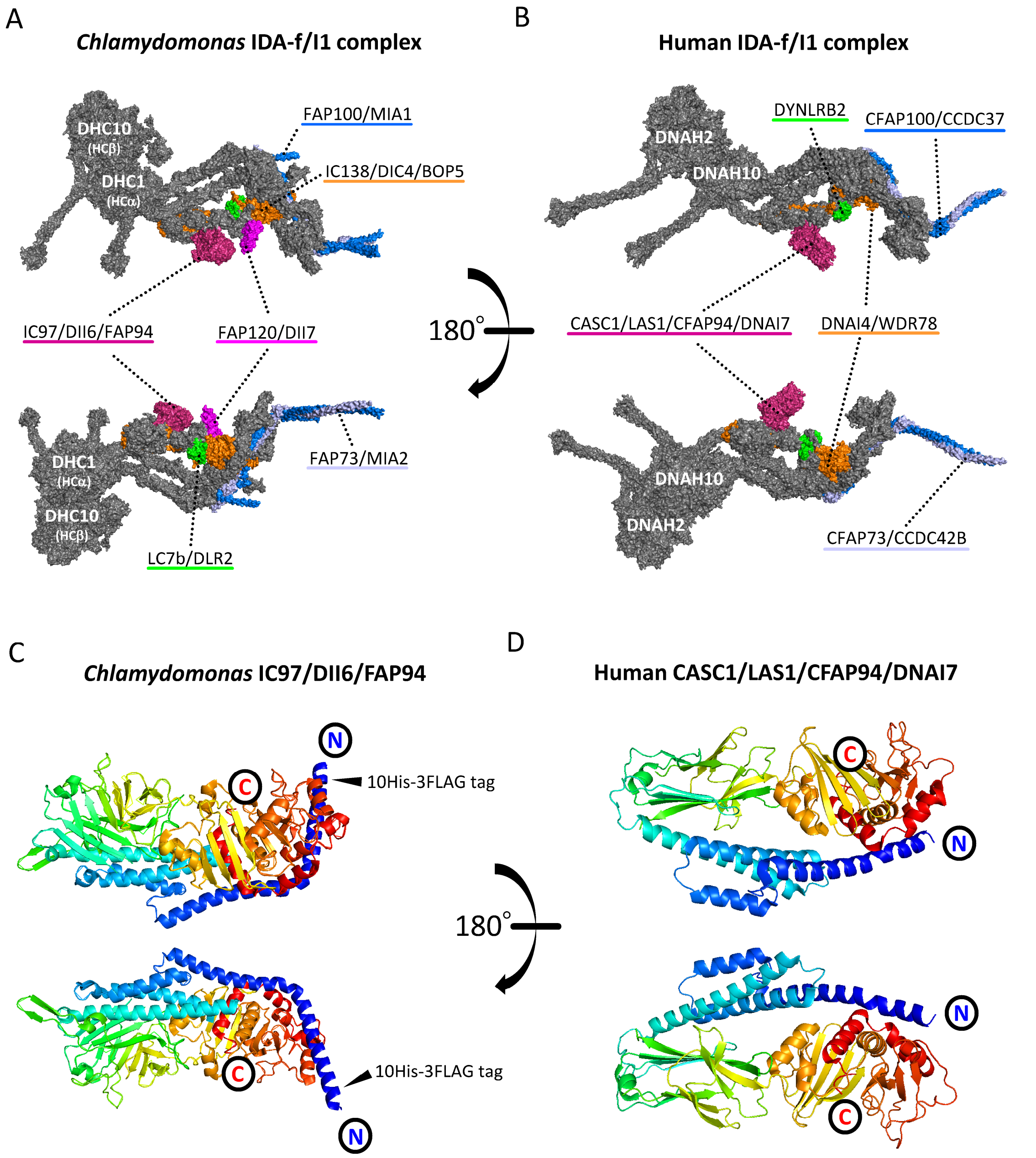

(A) A structural model of the *Chlamydomonas* IDA-f/I1 complex. The positions of IC138, IC97, FAP120, LC7b, FAP100/MIA1, and FAP73/MIA2 are highlighted. IC138, IC97, FAP120, and LC7b form a regulatory subcomplex of IDA f/I1 (1-4). FAP100/MIA1 and FAP73/MIA2 (the major components of the MIA complex), together with the N-terminal half of the FAP57 homodimer, form a docking-complex-like structure of IDA f/I1 that links IDA f/I1, doublet microtubule, ODA, and the N-DRC (4). **(B)** A structural model of human IDA-f/I1 complex. The positions of DNAI4 (IC138 ortholog), CASC1 (IC97

ortholog), DYNLRB2 (LC7b ortholog), CFAP100 (FAP100/MIA1 ortholog), and CFAP73 (FAP73/MIA2 ortholog) are highlighted. No obvious FAP120 ortholog was identified in human respiratory cilia (4). **(C)** A molecular model of the *Chlamydomonas* IC97 protein. The insertion site of the 10His-3FLAG tag in the rescued strain (*ic97; IC97:10His-3FLAG-TG*) is also shown. The predicted structure has an extended helix near the N-terminus. **(D)** A molecular model of the human CASC1 protein. In this figure, the models for **(A)** and **(C)** are taken from the PDB database (8GLV) (4), and the models for **(B)** and **(D)** are taken from the PDB database (8J07) (4). The structures were DSSP-refined, and the unmodeled residues/chains are not shown in this figure.

Supplemental Figure 2. Multiple alignment of *Chlamydomonas* IC97 and its potential orthologs.

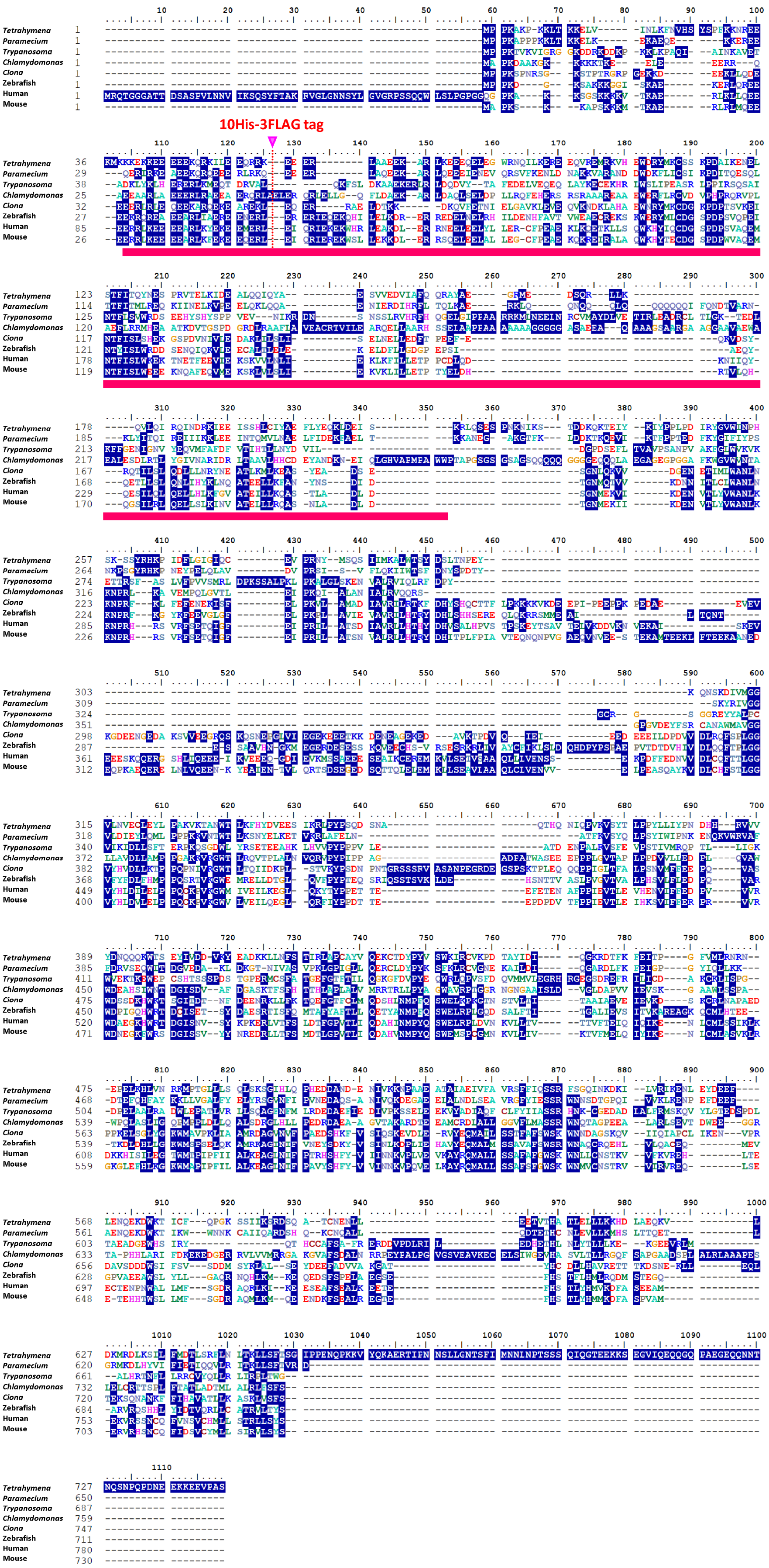

Chlamydomonas IC97 is evolutionarily conserved in eukaryotes from lower unicellular microorganisms to higher multicellular organisms. The *Chlamydomonas* IC97 sequence and the sequences of its potential orthologs were aligned using the Clustal Omega program (https://www.ebi.ac.uk/jdispatcher/msa/clustalo) (5), and the output file was edited using BioEdit (https://thalljiscience.github.io/) (6). The insertion site of the 10His-3FLAG tag in the rescued strain (*ic97; IC97:10His-3FLAG-TG*) is shown as a dotted red line. The position of the Casc1_N domain in *Chlamydomonas* IC97 is also shown as a bold reddish-purple line. The accession numbers of the sequences for this alignment are as follows: *Chlamydomonas reinhardtii* IC97 [NCBI: ACN22075.1 (2)], *Homo sapiens* CASC1 [NCBI: XP_054228387.1], *Mus musculus* CASC1 [NCBI: NP_796196.3], *Danio rerio* DNAI7 [NCBI: NP_001092924.1], *Ciona intestinalis* DNAI7 [NCBI: XP_018669746.1], *Trypanosoma brucei* DNAI7-like protein [NCBI: XP_844216.1], *Tetrahymena thermophila* DNAI7-like protein [TGD (https://tet.ciliate.org/): THERM_00437360], and *Paramecium tetraurelia* DNAI7-like protein [NCBI: XP_001431590.1].

Supplemental Table 1. *Chlamydomonas* mutant strains used in this study.

Strain Name	Mutated Protein/Description	Reference
bop5-3	IC138/DIC4/BOP5; lacking the IC138 sub-complex (IC138, IC97, FAP120, and LC7b) in flagella	(3, 7)
ic97 (Original)	IC97/DII6/FAP94; original CLiP strain of *ic97* (LMJ.RY0402.067788)	(8)
ic97 (E2)	IC97/DII6/FAP94; backcrossed mutant progeny from crosses of the wild type and original *ic97* (LMJ.RY0402.067788), used primarily for the phenotypic and biochemical analyses in this study, lacking IC97 and FAP120 in flagella	This study
ic97; IC97:10His-3FLAG-TG (D1Sd)	Rescued *ic97* (E2) strain expressing the epitope-tagged IC97 protein (IC97-10His-3FLAG), backcrossed once to wild type before use	This study
ida1	DHC1/IDA1/PF9 (IDA-f/I1 HCα); lacking IDA f/I1 in flagella	(9)
mia2-2	FAP73/MIA2; lacking one of the core proteins of the MIA complex and has hyperphosphorylated IC138	(10-12)
oda1	ODA-DC2/ODA1; lacking ODA in flagella	(13)

References

1. Bower R, VanderWaal K, O'Toole E, Fox L, Perrone C, Mueller J, Wirschell M, Kamiya R, Sale WS, Porter ME. 2009. IC138 defines a subdomain at the base of the I1 dynein that regulates microtubule sliding and flagellar motility. Mol Biol Cell 20:3055-63.
2. Wirschell M, Yang C, Yang P, Fox L, Yanagisawa HA, Kamiya R, Witman GB, Porter ME, Sale WS. 2009. IC97 is a novel intermediate chain of I1 dynein that interacts with tubulin and regulates interdoublet sliding. Mol Biol Cell 20:3044-54.
3. Ikeda K, Yamamoto R, Wirschell M, Yagi T, Bower R, Porter ME, Sale WS, Kamiya R. 2009. A novel ankyrin-repeat protein interacts with the regulatory proteins of inner arm dynein f (I1) of *Chlamydomonas reinhardtii*. Cell Motil Cytoskeleton 66:448-56.
4. Walton T, Gui M, Velkova S, Fassad MR, Hirst RA, Haarman E, O'Callaghan C, Bottier M, Burgoyne T, Mitchison HM, Brown A. 2023. Axonemal structures reveal mechanoregulatory and disease mechanisms. Nature 618:625-633.
5. Sievers F, Higgins DG. 2014. Clustal omega. Curr Protoc Bioinformatics 48:3.13.1-3.13.16.
6. Hall TA. 1999. BioEdit: a user-friendly biological sequence alignment editor and analysis program for Windows 95/98/NT. Nucleic Acids Symp Ser 41:95-98.
7. VanderWaal KE, Yamamoto R, Wakabayashi K, Fox L, Kamiya R, Dutcher SK, Bayly PV, Sale WS, Porter ME. 2011. *bop5* mutations reveal new roles for the IC138 phosphoprotein in the regulation of flagellar motility and asymmetric waveforms. Mol Biol Cell 22:2862-74.
8. Li X, Zhang R, Patena W, Gang SS, Blum SR, Ivanova N, Yue R, Robertson JM, Lefebvre PA, Fitz-Gibbon ST, Grossman AR, Jonikas MC. 2016. An indexed, mapped mutant library enables reverse genetics studies of biological processes in *Chlamydomonas reinhardtii*. Plant Cell 28:367-87.
9. Kamiya R, Kurimoto E, Muto E. 1991. Two types of *Chlamydomonas* flagellar mutants missing different components of inner-arm dynein. J Cell Biol 112:441-7.
10. Yamamoto R, Song K, Yanagisawa HA, Fox L, Yagi T, Wirschell M, Hirono M, Kamiya R, Nicastro D, Sale WS. 2013. The MIA complex is a conserved and novel dynein regulator essential for normal ciliary motility. J Cell Biol 201:263-78.
11. King SJ, Dutcher SK. 1997. Phosphoregulation of an inner dynein arm complex in *Chlamydomonas reinhardtii* is altered in phototactic mutant strains. J Cell Biol 136:177-91.
12. Kutomi O, Yamamoto R, Hirose K, Mizuno K, Nakagiri Y, Imai H, Noga A, Obbineni JM, Zimmermann N, Nakajima M, Shibata D, Shibata M, Shiba K, Kita M, Kigoshi H, Tanaka Y, Yamasaki Y, Asahina Y, Song C, Nomura M, Nomura M, Nakajima A, Nakachi M, Yamada L, Nakazawa S, Sawada H, Murata K, Mitsuoka K, Ishikawa T, Wakabayashi K-I, Kon T, Inaba K. 2021. A dynein-associated photoreceptor protein prevents ciliary acclimation to blue light. Sci Adv 7:eabf3621.
13. Kamiya R. 1988. Mutations at twelve independent loci result in absence of outer dynein arms in *Chlamydomonas reinhardtii*. J Cell Biol 107:2253-8.
